# Improvement of RTT Fairness Problem in BBR Congestion Control Algorithm by Gamma Correction

**DOI:** 10.3390/s21124128

**Published:** 2021-06-16

**Authors:** Wansu Pan, Xiaofeng Li, Haibo Tan, Jinlin Xu, Xiru Li

**Affiliations:** 1 Hefei Institute of Physical Science, Chinese Academy of Sciences, Hefei 230031, China; wansupan@mail.ustc.edu.cn (W.P.); hbtan@hfcas.ac.cn (H.T.); jlxu@hfcas.ac.cn (J.X.); xrli@hfcas.ac.cn (X.L.); 2 University of Science and Technology of China, No.96 JinZhai Road, Baohe District, Hefei 230026, China

**Keywords:** TCP congestion control, BBR, RTT fairness, gamma correction, pacing gain

## Abstract

Google proposed the bottleneck bandwidth and round-trip propagation time (BBR), which is a new congestion control algorithm. BBR creates a network path model by measuring the available bottleneck bandwidth and the minimum round-trip time (RTT) to maximize delivery rate and minimize latency. However, some studies have shown that there are serious RTT fairness problems in the BBR algorithm. The flow with longer RTT will consume more bandwidth and the flows with shorter RTT will be severely squeezed or even starved to death. Moreover, these studies pointed out that even small RTT differences will lead to the throughput of BBR flows being unfair. In order to solve the problem of RTT fairness, an improved algorithm BBR-gamma correction (BBR-GC) is proposed. BBR-GC algorithm takes RTT as feedback information, and then uses the gamma correction function to fit the adaptive pacing gain. This approach can make different RTT flows compete for bandwidth more fairly, thus alleviating the RTT fairness issue. The simulation results of Network Simulator 3 (NS3) show that that BBR-GC algorithm cannot only ensure the channel utilization, but also alleviate the RTT fairness problem of BBR flow in different periods. Through the BBR-GC algorithm, RTT fairness is improved by 50% and the retransmission rate is reduced by more than 26%, compared with that of the original BBR in different buffer sizes.

## 1. Introduction

With the development of network communication, the performance requirements of users for network communication need to be improved. On the one hand, scholars such as Tsiropoulou E.E. et al. [1,2] using the game theory method proposed to solve the problem of power and rate allocation. These make the configuration mechanism of network resources optimized, and then improves the performance of network communication. On the other hand, network congestion control is also very important for the improvement of network performance. However, with the increasing demand of network applications, the performance of rate control algorithm based on AIMD is poor [3,4]. Once packet loss occurs, the traditional AIMD algorithm will consume quite a long time to recover to the congestion window (CWND). Similar to the AIMD algorithm, some scholars propose Reno [5], BIC [6], CUBIC [7] and other congestion control algorithms (CCAs) to adjust the increase or decrease behavior of CWND through packet loss feedback, so as to adapt to the high-speed network. However, in the network environment with link packet loss, the throughput drops seriously, and a lot of bandwidth is often wasted.

In 2016, Google released bottleneck bandwidth round trip propagation time (BBR) algorithm [8,9,10]. Unlike packet loss and delay as congestion indicators in loss-based CCAs, BBR adjusts its sending behavior according to the estimated bottleneck bandwidth (Btlbw) and round-trip propagation time (RTprop) to achieve high throughput and minimum transmission delay. The goal of BBR is to run at the Kleinrock’s optimal operating point [11], that is, the inflight data are equal to a bandwidth delay product (BDP). The implementation of BBR published by Google shows that the BBR’s throughput of TCP connection is obviously higher than that of CUBIC. BBR has received a lot of attention since it has been released. Literature [12,13,14] has evaluated the behavior of BBR and proposed some problems: unfairness in sharing bottleneck between BBR and Reno/CUBIC, the RTT fairness problem of BBR, massive packets retransmission and so on. Aware of the problem of BBR algorithm, some scholars have modified the regulation factors of BBR to improve the defects of BBR itself [15,16,17]. On the other hand, BBR is applied to different network domains to achieve a better performance [18,19,20,21]. Google is also constantly optimizing BBR algorithm and updating BBR v2 version [22] to improve coexistence with other algorithms and reduce the attack. In the latest meeting [23], BBR.Swift method was introduced in the update of BBR v2. Some researchers evaluated and validated BBR v2 alpha. Kafoury et al. [24] evaluated the BBR v2 alpha version [25] and found that RTT fairness still existed.

Because BBR is still in the development stage, fairness between BBR algorithm and other loss-based CCAs [26,27,28,29] and RTT fairness [12,30,31,32,33,34] need to be improved. In the above analysis, one of the most serious problems of BBR is the RTT fairness problem in the protocol. When multiple flow shares have bottleneck links, BBR tends to favor long RTT flow [12]. Compared with shorter RTT flows, longer RTT flows can achieve higher throughput and transmission rate. What is more serious, is that short RTT flows will suffer from serious packet loss and high retransmission after it is compressed by long flows. Some users may exploit this vulnerability to maliciously compete for the precious bandwidth.

In order to alleviate the RTT fairness, Ma et al. [12] analyzed the reasons for the RTT fairness of BBR and proposed a BBQ algorithm. By limiting the probe phase of long RTT flow, the number of packets sent by long RTT flow is reduced. Tao et al. [30] proved that the RTT differences between BBR flows will affect the bandwidth occupation. When the RTT ratio is greater than 2, the bandwidth utilization of flows is obviously unfair. Yang et al. [31] proposed a method to adaptively adjust BBR sending rate by using link state, which is called adaptive BBR algorithm. Kim [30] et al. proposed Delay-Aware BBR (DA-BBR) algorithm to alleviate the RTT fairness problem, which reduced the BDP based on the RTT. Sun et al. [33] put forward the RFBBR algorithm to improve the RTT fairness of BBR, which could adjust the CWND value according to the different states in the queue. In our previous study [34], we proposed that the CWND could be adjusted by Btlbw and RTT to mitigate the RTT fairness. These above-mentioned algorithms have achieved some results in improving the fairness of RTT. Obviously, new methods are still needed to further improve the RTT fairness and practicability of BBR algorithm.

We tested the BBR algorithm on Network simulator 3(NS3) based on BBR implementation version [35,36,37,38]. Through a lot of analysis, we found that BBR uses pacing gain of 1.25 or 0.75 to make inflight of respective flow increases by 1.25 times in probe up phase, and then in the probe down phase inflight falls back to its initial value. In this process, extra packets cannot be released completely, and a persistent queue will be created in the bottleneck. The estimated BDP value of long RTT flow is larger than that of short flow, so the proportion of long flow in persistent queue is larger. Each BBR flow circulates this process repeatedly, so the longer flows gradually occupies the bandwidth advantage, forming an unfair global synchronization. As the RTT ratio between BBR flows increases, RTT fairness deteriorates. Therefore, we can use the RTT of each BBR flow to calculate the gain coefficient during the detection bandwidth period, instead of the fixed 1.25 or 0.75, so as to alleviate the unfairness between different RTT flows.

On the basis of the above discussion, this paper proposes an optimized algorithm based on BBR, named BBR-GC. The BBR-GC algorithm adjusts the pacing gain of different flows through gamma correction, to keep the data of different RTTs flows in the bottleneck queue roughly the same. NS3 is used to compare the BBR algorithm, BBQ algorithm, BBR-ACW algorithm and BBR-GC algorithm in different simulation environments, and the simulation results show that BBR-GC algorithm has a better performance. Compared with the original BBR, the RTT fairness of BBR-GC was improved by 50%, the retransmission rate was reduced by more than 26%, and the latency was also reduced by 57%.

The rest of this article is arranged as follows. Section 2 introduces the algorithm of BBR and analyzes RTT fairness issues. The theoretical model and derivation of BBR-GC algorithm are in Section 3. Section 4 is simulation results and evaluation. Conclusions and discussions are held in Section 5.

## 2. Background

### 2.1. BBR Behavior Analysis

Unlike loss-based CCAs, BBR measures the maximum delivery rate and minimum transmission delay alternately to find the Kleinrock’s optimal operating point [11].

BBR controls congestion by limiting the pacing rate of packets, limiting inflight to one BDP (BDP=Btlbw×RTprop). BBR adjusts the speed of output packets at the latest estimated delivery rate. At the same time, BBR maintains CWND in order to maintain consistent throughput in delayed or aggregated ack networks. BBR regards the maximum bandwidth (delivery rate) of the last 10 RTTs as the current Btlbw, and regards the minimum delay measured in the past 10 s as the current RTprop. Through the scaling factor cwnd_gain and pacing_gain to adjust CWND and pacing rate, as shown in Equations (1) and (2).

(1) pacing rate(sending rate)=pacing_gain×Btlbw

(2) CWND=cwnd_gain×BDP

**Figure 1:**
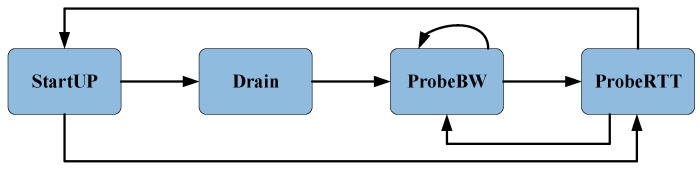
The phases of BBR.

The BBR algorithm has four control states: StartUP, Drain, ProbeBW and ProbeRTT. The Phases of BBR is shown in Figure 1.

In the StartUP phase, pacing rate and CWND will increase by setting cwnd_gain and pacing_gain as 2/ln2 (about 2.89). The exponential growth of pacing rate and CWND will lead to queue accumulation on routers. If the newly estimated bandwidth of three consecutive RTTs does not increase by at least 25%, the BBR enters the Drain phase. In the Drain phase, BBR passes the pacing_gain is reduced to ln2/2 (about 0.35) to clear the remaining queue in the previous stage, cwnd_gain remains unchanged (2/ln2). At the end of this phase, inflight data < the estimated BDP. In the ProbeBW phase, BBR has eight cycles in the detection bandwidth (pacing_gain []=[1.25; 0.75; 1; 1; 1; 1; 1; 1; 1; 1]), and the duration of each pacing_gain was RTprop. In the probe up cycle, pacing_gain = 1.25 is used to detect more bandwidth. In the next probe down cycle, pacing_gain = 0.75 is used to release the created queue. Then, in the next six cycles, pacing_gain = 1 is used to set the sending rate of BBR to Btlbw. At this phase, cwnd_gain is set to a fixed value of 2, which means that the upper flight limit is fixed at 2 BDP. If new RTprop is not sampled again within 10 s, BBR enters ProbeRTT. In this phase, the CWND is set to 4MSS and lasts for 200 ms.

### 2.2. BBR’s RTT Fairness

BBR calculates the pacing rate by active measurement behavior, which is performed on each end-to-end host, so bandwidth detection between different RTT flows is independent. The fairness of BBR must be guaranteed by each flow itself, and it is difficult for each independent flow to share bandwidth fairly.

We further analyze the ProbeBW phase, flows through the pacing_gain = 1.25 times increases their inflight by 1.25 times in the probe up phase. Then, in the probe down phase, the pacing_gain = 0.75 makes inflight fall back to their respective initial values. Finally, the pacing_gain = 1 for the remaining 6 cycles to maintain stable bandwidth. When only one stream passes through the bottleneck link, the upper limit of the transfer rate is Btlbw after the StartUP phase. The BBR flow will converge at the Kleinrock’s point and run with the maximum delivery rate and minimum delay. When multiple flows in a bottleneck link, the total transmission rate is greater than the bottleneck bandwidth. The probe down phase is not enough to consume the queue formed on the bottleneck, which will lead to the formation of persistent queue backlog. The BBR flow will run at the point to the right of Kleinrock’s operation point. According to queuing theory, queue sharing determines flow’s throughput. Due to queue generation, short RTT flows are first limited by CWND, and additional bandwidth cannot be obtained even if more probes are performed.

On the other hand, the long RTT flows have a larger estimated BDP value, so the larger the proportion of persistent queue, the larger the bandwidth consumption. Due to the extrusion of the long RTT flow, the short RTT flow will reduce the delivery rate in the next bandwidth detection period. Through this loop, the pacing rate of short RTT flows become smaller, resulting in serious bandwidth degradation. Even if the free bandwidth is transferred, it will be rapidly preempted by other flows and cannot make up for the bandwidth loss. In summary, when the bottleneck link is overloaded, the long RTT flows can achieve a higher transmission rate than its short RTT flows. Moreover, the greater RTT radio between the two BBR flows, the worse RTT fairness. Some users may get high bandwidth by increasing the RTT maliciously. Therefore, the RTT fairness issue in BBR needs to be solved.

## 3. The Proposed Algorithm: BBR-GC

### 3.1. Design Motivation

The RTT fairness problem of BBR flows has two aspects: one is how to guarantee the fairness of competition when there is new free bandwidth (such as flow exiting the network); the other is how to make different RTT flows share bandwidth fairly when there is no free bandwidth to occupy (in a stable state: continuously sharing 100% bandwidth utilization).

Through the above analysis, in order to make different RTT flows compete fairly, each independent BBR flow needs to form a unified view of bandwidth distribution to guide their fair sharing of bandwidth. When the lower bandwidth occupied by BBR flows, the probe up coefficient and probe down coefficient are larger. In this way, the flow will greatly increase the bandwidth, slightly reduce the bandwidth, and occupy the effective bandwidth as soon as possible. On the contrary, when the higher bandwidth occupied by BBR flows, the probe up coefficient and probe down coefficient are smaller. Thus, the flows will increase bandwidth slightly, greatly reduces the bandwidth, and provides redundant bandwidth for other flows. Ideally, we can use the absolute value of pacing rate to calculate the coefficient of detection bandwidth period, thus replacing the fixed values of 1.25 and 0.75. Suppose that there are two different RTT flows in the link and the pacing rate of the long flow and the short flow is r_1_ and r_2_, respectively, after normalization, and the probe up coefficient as *P*_up_ and the probe down coefficient as *P*_down_. The *P*_up_ and *P*_down_ of long flow are $\frac{r_{1}+1}{r_{1}}$ and $\frac{r_{2}-1}{r_{2}}$, and the *P*_up_ and *P*_down_ of the short flow are $\frac{r_{2}+1}{r_{2}}$ and $\frac{r_{1}-1}{r_{1}}$, respectively. In this way, the *P*_up_ and *P*_down_ of two flows can be interleaved with each other. If the pacing rate of a flow is large, it will probe up slowly and draw down quickly. If the pacing rate of a flow is small, it will probe up quickly and draw down slowly. By flexibly pacing gain regulation, different RTT flows can share bandwidth fairly in any case. However, the BBR measures the pacing rate independently, and there are more than two flows in the actual network. This makes the above method unapplicable in the actual process. Therefore, we try to find a global variable to obtain ideal *P*_up_ and *P*_down_, and construct a feedback model about the pacing gain. It can be applied to multiple BBR flows of different RTTs, and improve RTT fairness without affecting the bandwidth utilization.

Algorithm 1 describes the implementation logic of our improved algorithm BBR-GC. When in probe up phase, if inflight < 1 BDP, different RTT flows actively detect bandwidth according to their *P*_up_. When the link state changes to inflight >1 BDP, the inflight data will exceed the bottleneck transmission capacity and form a queue in the bottleneck buffer, which will be adaptively adjusted according to different RTT flows to reduce the inflight data. When in probe down phase, if the link state changes to inflight >1.25 BDP and packet loss occurs (or has loss indication), the number of packets entering the pipeline in the next cycle is reduced according to the coefficient *P*_down_.
**Algorithm 1: ProbeBW phase in BBR-GC****Input**: rtt_us, inflight, has loss//The value of rtt_us is updated when an ACK is received **Output**: pacing gain 1:  RTTmin = min (RTTmin, rtt_us); 2.  RTTmax = max (RTTmax, rtt_us); **Phase 1: probe up** 3.  **For** every ACK **do** 4:  **if** inflight < 1BDP **then** 5:   pacing_gain = **P_up_** 6:   **return** 7:  **end if** 8:  **if** pacing_gain < 1.0 **and** inflight < 1BDP **then** 9:   pacing_gain = 1.0 10:   **end if** **Phase 2: probe down** 1:  **if** inflight > 1.25BDP **or** has loss **then** 2:   pacing_gain = **P_down_** 3:  **return** 4:  **end if** **Phase 3: next six cycles** 1:  **if** pacing gain == 1.0 **then** 2:  **return** 3:  **end if**

Algorithm 1 gives the pseudo code of BBR-GC, which is an improvement of BBR algorithm. The algorithm complexity analysis uses the big O notation [39,40], which can be divided into time complexity and space complexity. This method uses another function (usually simpler) to describe the asymptotic upper bound of the order of magnitude of a function. The number of statements executed in an algorithm is called statement frequency or time frequency, denoted as T(n). If there is an auxiliary function f(n) such that when n approaches infinity, the limited value of T(n)/f(n) is a constant not equal to zero, then f(n) is said to be a function of the same order of magnitude of T(n). Let T(n) = O(f(n)), O(f(n)) is the time complexity of the algorithm. Similar to the discussion of time complexity, the space complexity S(n) of an algorithm is defined as the storage space consumed by the algorithm, which is also a function of the problem size n. Let S(n) = O(f (n)), O(f(n)) be the space complexity of the algorithm.

We refer to the analysis of the complexity of the congestion control algorithm in literature [20]. The time complexity of the algorithm reflects the magnitude of the increase of program execution time with the increase of input size. The BBR-GC algorithm adopts if loop statement to judge state condition, so the time complexity of its algorithm is O(1). The space complexity of the algorithm represents the growth relationship between data size and storage space. Since each cycle of the BBR-GC algorithm only stores the results of pacing gain operation without additional memory consumption, the space complexity of the BBR-GC algorithm is also O(1). In addition, the implementation of BBR-GC algorithm is still based on the original BBR framework, and BBR-GC is implemented into NS3 as a Linux-based module. This method provides a good preparation for the implementation of the Linux kernel and facilitates the implementation and extension of the BBR-GC algorithm.

### 3.2. Algorithm Model Analysis

In order to better describe the relationship between various parameters of the BBR algorithm, we carry out modeling analysis on the BBR algorithm. Suppose that there are *n* different flows passing through the bottleneck link with the bandwidth of *C*, let $flow_{i}(i\in \left[1;\;n\right])$ denote the flow and $d_{i}$ denote the delivery rate. According to the definition of BBR algorithm, we can get the estimated bandwidth of flows at t time, which can be calculated by (3).

(3) $${\mathrm{Btlbw}}_{i}\left(t\right)=max\left(d_{i}(T)\right)\left(T\in \left[t-10RTT;\;t\right]\right)$$

Due to the generation of the queue, the round-trip time of $flow_{i}$ at time t can be calculated by (4):

(4) $$T_{i}(t)=q_{i}(t)+\mathrm{RTprop}$$

where $q_{i}(t)$ denotes the queuing delay. We use $I_{i}(t)$ denote inflight data at time t, which can be obtained by $d_{i}\left(t\right)$ as shown in (4).

(5) $$I_{i}\left(t\right)=d_{i}\left(t\right)\times T_{i}$$

We assume that the two different RTTs BBR flows send data from different source nodes. The RTT of flow1 and flow2 are set to $T_{1}$ and $T_{2}$, respectively, and $T_{2}=aT_{1}(a\geq 1)$. The total inflight data in the time interval [*t*, *t* + a] of the two flows can be calculated by (6) and (7).

(6) $$I_{1}\left(t\right)=\left[d_{1}\left(\alpha \right)\times at+d_{1}(\alpha -1)\times (1-at)\right]\times T_{1}$$

(7) $$I_{2}\left(t\right)=d_{2}\left(t-1\right)\times T_{2}$$

where α=⌊(t−1)×a⌋. The bandwidth occupation of different flows in the link can be determined by $I_{i}(t)$:

(8) $$oBw_{i}\left(t\right)=C\times \frac{I_{i}\left(t\right)}{\sum_{i=0}^{n}I_{i}(t)}$$

Substituting (6) and (7) into (8), we have:

(9) $$oBw_{2}\left(t\right)=C\times \frac{I_{2}(t)}{I_{2}(t)+I_{1}(t)}=C\times \frac{a\times d_{2}(t-1)}{a\times d_{2}(t-1)+I_{1}(t)}$$

It can be seen from (9) that the bandwidth occupation of flow2 is related to ratio a. When $d_{i}$ increases, $oBw_{2}$ also increases, which means that flow2 preempts the bandwidth of flow1.

To sum up, the throughput of RTT flows is affected by the ratio between RTTs [33]. We can use $T_{i}$ to reflect the inflight data of the link and construct a negative feedback model to constrain *P*_up_ and *P*_down_. The BBR flow with lower bandwidth occupancy is expected to have larger *P*_up_ and *P*_down_. On the contrary, BBR flow with the higher the bandwidth occupancy is expected to have the smaller *P*_up_ and *P*_down_. Let $\omega_{i}$ be defined as the percentage of current delay and maximum delay:

(10) $$\omega_{i}=\frac{T_{i}}{T_{max}}\left(\omega_{i}\in (0,1]\right)$$

where $T_{max}$ is the maximum $T_{i}$ over this connection and where $\omega_{i}=1$ only when the bottleneck link capacity and buffer are fully occupied. It can be seen from Equation (10) that the larger $T_{i}$ is, the greater $\omega_{i}$ is.

We propose an improved algorithm by using RTT feedback, called BBR-GC. BBR-GC adjust *P*_up_ and *P*_down_ in ProbeBW phase according to $\omega_{i}$ reaction link state. For the *P*_up_, the correlation function $P_{\mathrm{up}}(\omega_{i})$ should create a concave downward curve, and the lower asymptote is *P*_up_ = 1. It can reduce the bandwidth occupation of the dominant flows and provide more available bandwidth for the vulnerable flows. When the current $\omega_{i}$ is larger, the function $P_{\mathrm{up}}(\omega_{i})$ should decline slowly. The function $P_{\mathrm{up}}(\omega_{i})$ should decline actively when the $\omega_{i}$ coefficient is smaller. For the *P*_down_, the correlation function $P_{\mathrm{down}}(\omega_{i})$ should be an upper convex curve, and the upper asymptote is *P*_down_ = 1. When $\omega_{i}$ is larger, the function $P_{\mathrm{down}}(\omega_{i})$ should decline rapidly. The function should decline slowly when $\omega_{i}$ is smaller. Moreover, the required function must be a low complexity function, because it needs to be implemented in BBR algorithm. Therefore, based on the above constraints, we construct two functions $P_{\mathrm{up}}(\omega_{i})$ and $P_{\mathrm{down}}(\omega_{i})$, respectively. We test some functions and find that the gamma correction function can meet the needs. By adjusting the pacing gain through gamma correction, the long flow’s pacing rate is limited, which limits the bandwidth occupation of the long flow strictly. The bandwidth detection ability of the short flow is improved, so that different RTT flows can compete more fairly.

In image processing, gamma correction is used to smooth out the details of the tone [41]. The Equation is as follows (11):

(11) $$f(I)=I^{\gamma }$$

**Figure 2:**
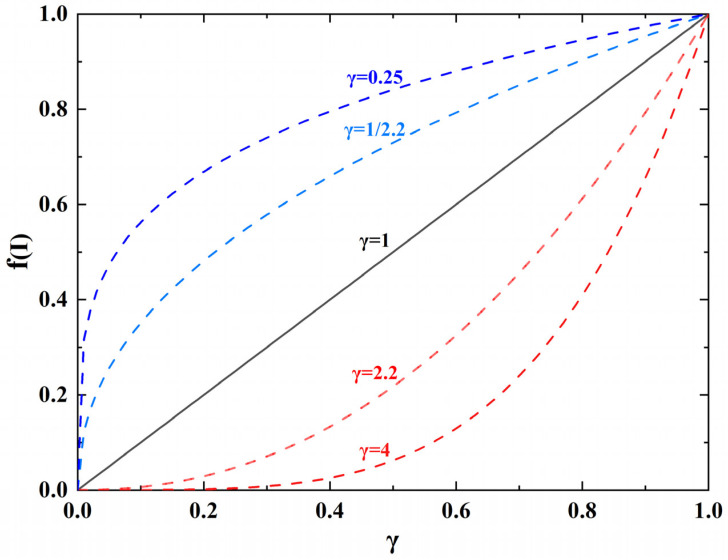
The curve of gamma correction.

The gamma correction curve is shown in Figure 2. When γ<1, as shown by the blue dotted line, the dynamic range is large in the low gray value region. In the region of high gray value, the dynamic range is small. When γ>1, as shown by the red dotted line, the dynamic range is small in the low gray value region. In the region of high gray value, the dynamic range is large. Gamma correction achieves enhanced image contrast through histogram weighted average, equalization, correction, and combination of the original image [42,43]. This enhancement technology plays an important role in digital image processing, computer vision and pattern recognition. Therefore, we want to adjust the packing gain by gamma correction. By changing the parameters of gamma correction function, we can meet the actual needs of $P_{\mathrm{up}}(\omega )$ function and $P_{\mathrm{down}}(\omega )$ function. As Equations (12) and (13) shows:

(12) $$P_{\mathrm{up}}(\omega )=1.5-0.5\times \omega^{0.25};\;\left(P_{\mathrm{up}}(\omega )\in [1,1.5]\right)$$

(13) $$P_{\mathrm{down}}(\omega )=1-0.5*\omega^{4};\;\left(P_{\mathrm{down}}(\omega )\in [0.5,1]\right)$$

**Figure 3:**
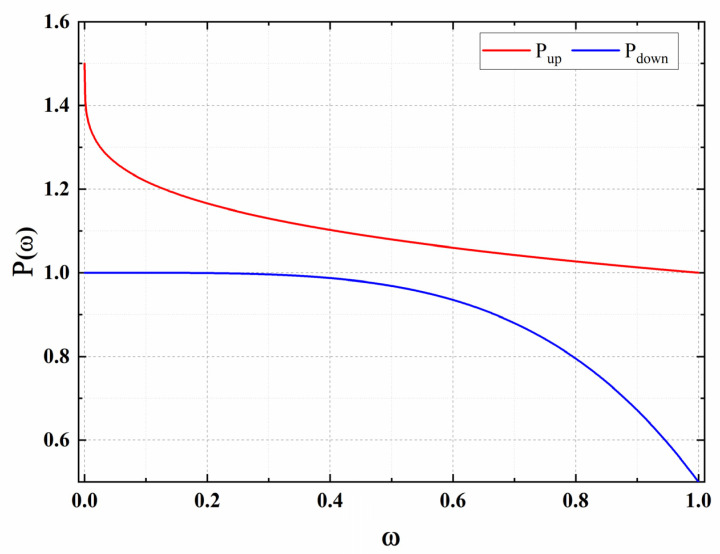
The curve of *P*_up_ and *P*_down_ functions.

The change trend of $P_{\mathrm{up}}(\omega )$ and $P_{\mathrm{down}}(\omega )$ is shown in Figure 3. With the increase of ω, $P_{\mathrm{up}}(\omega )$ decreases slowly, and the decreasing trend becomes slower. The value range of $P_{\mathrm{up}}(\omega )$ is between 1 and 1.5. With the increase of ω, the $P_{\mathrm{down}}(\omega )$ decreases slowly and the decreasing trend becomes faster, and the value range of $P_{\mathrm{down}}(\omega )$ is between 0.5 and 1.

BBR-GC further enhances the ability of network state regulation through this feedback regulation of pacing gain. The bandwidth of each BBR flow is balanced by the *P*_up_ and the *P*_down_. On one hand, we extend the upper limit of *P*_up_ to 1.5, which makes the detection bandwidth stage of BBR much faster. On the other hand, the lower limit of *P*_down_ is set to 0.5, which makes the fast recovery stage of BBR much faster. The probe up and probe down coefficients of each flow are staggered with each other by dynamic adjustment. Therefore, compared with the original BBR, BBR-GC can ensure the bandwidth utilization rate, and make the pacing rate of each flow converge to the fairness center and improve the fairness of BBR.

## 4. Results and Evaluation

**Figure 4:**
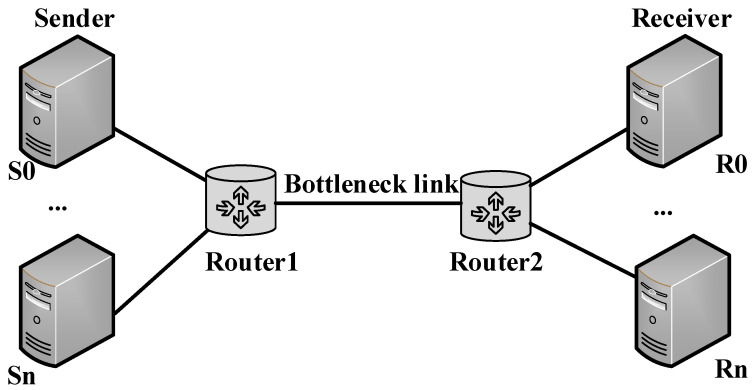
Experimental network topology.

Different from the traditional congestion control algorithm, BBR prefers large RTT flows and allocates more bandwidth for large RTT flows. This bias will make a trade-off between low latency and high transmission rate, breaking the concept of finding the optimal operating point with the minimum RTT. This study proposes an optimized algorithm based on the original BBR, named BBR-GC, to improve the RTT fairness of BBR without affecting the bandwidth utilization of the network. In order to evaluate the performance of BBR-GC, the original BBR algorithm, the BBQ algorithm and the BBR-ACW algorithm are introduced as the benchmark. The BBQ algorithm improves RTT fairness by setting an upper limit on the span of detection cycle and reducing the detection time of flows with long RTT. The BBR-ACW algorithm solves the limitation of CWND by adjusting cwnd_gain, so as to alleviate the RTT fairness issue. We use NS3 to do a lot of simulation experiments, and compare the fairness between BBR, BBQ, BBR-ACW and BBR-GC. The experimental topology is shown in Figure 4. This section describes the results of running tests under different network conditions.

### 4.1. RTT Fairness

**Figure 5:**
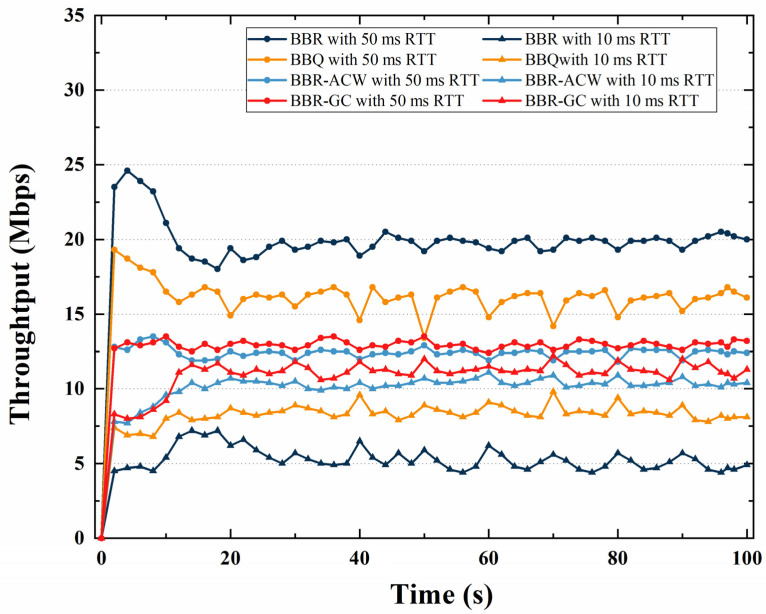
The throughput comparison of BBR, BBQ, BBR-ACW, and BBR-GC algorithms with different RTTs.

The comparison of link fairness with different RTTs or using different CCAs is mainly carried out by comparing the throughput occupied by each link. The throughput of BBR, BBQ, BBR-ACW and BBR-GC is compared through simulation experiments, and performance of BBR-GC is evaluated. In the simulation experiment, we set the bottleneck bandwidth as 100 Mbps and the buffer size as 5 BDP. The RTT fairness is evaluated by comparing the throughput of 10 ms flow and 50 ms flow. The throughput comparison of BBR, BBQ, BBR-ACW, and BBR-GC algorithms with different RTTs is shown in Figure 5.

From Figure 5, we can see that regardless of the BBR, BBQ, BBR-ACW, or BBR-GC, the larger RTT is, the larger the throughput will be. For BBR algorithms, the throughput of a 50 ms RTT flow is 4 times that of a 10 ms RTT flow. The throughput difference between two BBQ flows with different RTTs is 1.6 times. For BBR-ACW and BBR-GC algorithms, the throughput of a 50 ms RTT flow is about 1.2 times that of a 10 ms RTT flow. BBQ, BBR-ACW, and BBR-GC have better bandwidth allocation than BBR, and increase the bandwidth occupancy ratio of 10 ms RTT. However, compared with the BBR and BBQ algorithms, BBR-GC has the smallest throughput difference and the highest fairness. Compared with BBQ algorithm, BBR-GC algorithm suppresses the bandwidth of long RTT flows and improves the bandwidth of short RTT flows. Compared with BBR-ACW algorithm, the fairness of BBR-GC algorithm is close to that of BBR-ACW algorithm, but BBR-GC improves the throughput of flows.

Furthermore, to quantify the difference of RTT fairness problem of BBR algorithm in different buffer sizes, the Jain’s fairness index [44] is introduced. The Jain’s fairness index is used to measure the fairness of bandwidth allocation in the competition of bandwidth resources. The calculation method is shown in Equation (14).

(14) $$J=\left(\frac{(\sum_{i=1}^{n}x_{i}{)}^{2}}{n\sum_{i=1}^{n}x_{i}{}^{2}}\right)$$

The closer Jain’s fairness index is to 1, the better the fairness of bandwidth allocation is. Jain’s fairness index can well reflect the throughput difference.

**Figure 6:**
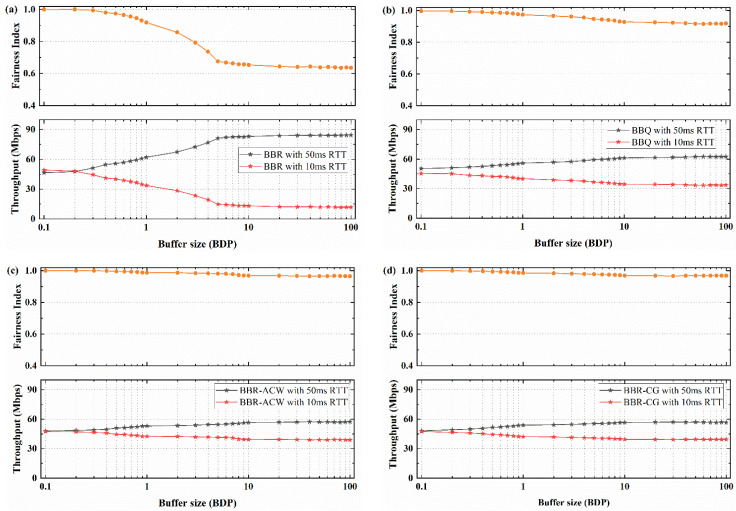
The average throughput and fairness index when 10 ms RTT flows competing with 50 ms RTT flows in different buffer size: (**a**) BBR; (**b**) BBQ; (**c**) BBR-ACW; (**d**) BBR-GC.

A large number of experiments are carried out to compare the differences between 10 ms RTT and 50 ms RTT flows in different buffer sizes, in order to further evaluate the fairness of the three algorithms. The average throughput and fairness index of different algorithms in different buffer size are shown in Figure 6.

Figure 6a shows the throughput change and fairness index when 10 ms RTT flow competes with 50 ms RTT flow of BBR algorithm. With the increase of buffer size, the throughput difference between 10 ms RTT flow and 50 ms RTT flow becomes larger. When the buffer size is less than 0.2 BDP, different RTT flows can share bandwidth fairly. The difference between 10 ms RTT flows and 50 ms RTT flows is less than 5 Mbps, and the fairness index is about 0.99. When the buffer is larger than 0.2 BDP, the bandwidth difference between the two flows increases with the increase of the buffer size. When the buffer is larger than 6 BDP, the throughput of 50 ms RTT is about 84.3 Mbps, and throughput of 10 ms RTT is only about 11.7 Mbps. The fairness index is only about 0.635.

In Figure 6b, BBQ algorithm can reduce the bandwidth difference between 10 ms RTT flows and 50 ms RTT flows. The bandwidth difference between the two flows increases with the increase of the buffer size, and the 50 ms RTT flows are always dominant. Compared with BBR algorithm, BBQ can improve the RTT fairness. When the buffer is larger than 10 BDP, the bandwidth occupancy ratio remains stable, and the bandwidth of 50 ms RTT is 62.5 Mbps. The fairness index of BBQ can maintain above 0.916.

As shown in Figure 6c, BBR-ACW algorithm can improve the RTT fairness between 10 ms RTT flows and 50 ms RTT flows. Although the throughput of 50 ms RTT flows is always dominant, maintaining a bandwidth size of 58.9 Mbps, it is only 1.2 times the bandwidth of 10 ms RTT flows. Compared with BBR algorithm and BBQ algorithm, the fairness index of BBR-ACW can maintain above 0.964.

For BBR-GC algorithm in Figure 6d, two flows can share bandwidth fairly. When the buffer size is less than 0.4 BDP, the fairness of BBR-GC is similar to that of BBQ and BBR-ACW, and the fairness index is approximately 1. When the buffer size is greater than 10 BDP, 50 ms RTT still accounts for most of the bandwidth of the play at about 56.9 Mbps, and the throughput of 10 ms RTT is improved to about 39.4 Mbps. In the buffer size distribution of 0.1 BDP to 100 BDP, the fairness index of BBR-GC is basically the same as that of BBR-ACW, which can be maintained above 0.968.

Overall, BBR-GC algorithm has better fairness than BBR algorithm and BBQ algorithm in different buffer size, and slightly better than that of BBR ACW algorithm. Especially in deep buffer, compared with BBR, the RTT fairness has been greatly improved, and the fairness index has been increased by 50%.

**Figure 7:**
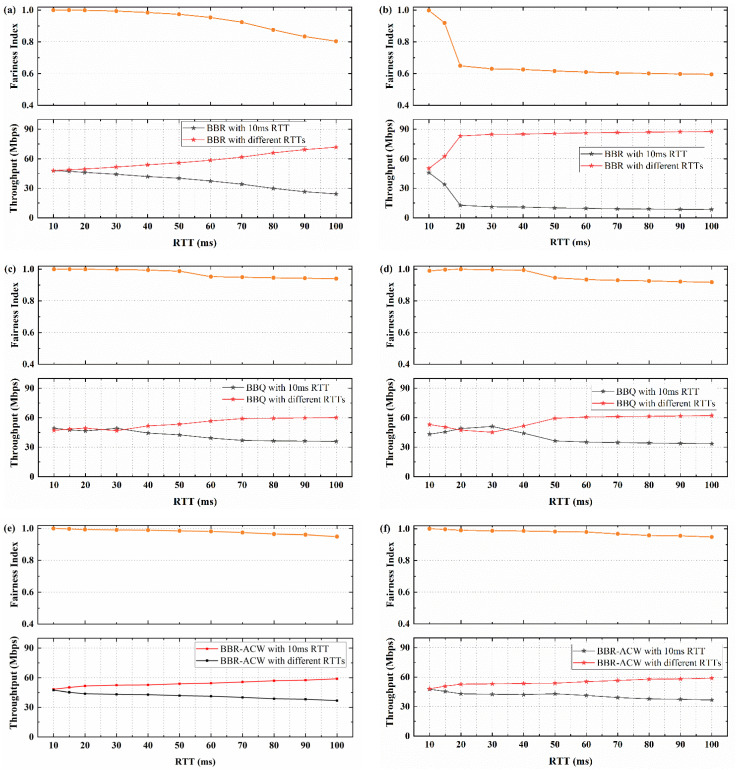

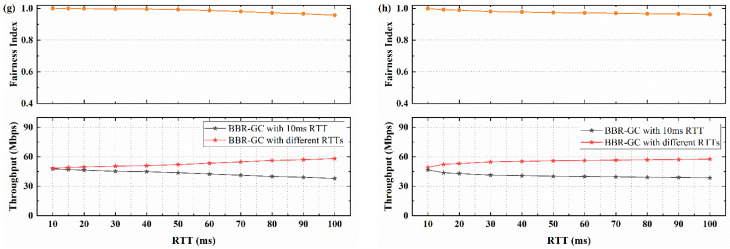
The average throughput and fairness index of flow when 10 ms RTT flows coexists with different RTTs flows: (**a**) BBR with 0.5 BDP buffer; (**b**) BBR with 5 BDP buffer; (**c**) BBQ with 0.5 BDP buffer; (**d**) BBQ with 5 BDP buffer; (**e**) BBR-ACW with 0.5 BDP buffer; (**f**) BBR-ACW with 5 BDP buffer; (**g**) BBR-GC with 0.5 BDP buffer; (**h**) BBR-GC with 5 BDP buffer.

We further conducted the hybrid experiments for different RTT flows to study the fairness of the four algorithms with different RTT ratios. The fairness changes with the change of RTT ratio in different buffer sizes. The effectiveness of BBR-GC algorithm was analyzed by comparing the bandwidth allocation and fairness index between10 ms RTT flows with different RTTs flows. The comparison of throughput variation and fairness index are shown in Figure 7.

Figure 7a shows the throughput change and fairness index when 10 ms RTT flow competes with different RTTs flow of BBR algorithm in 0.5 BDP buffer. When the RTT difference more than twice, the long RTT BBR flow occupies 70% bandwidth, and the fairness index of BBR is about 0.803. When the buffer size is increased to 5 BDP, as shown in Figure 7b. The bandwidth fairness of BBR decreases with the increase of RTT difference. The throughput of 20 ms RTT flows is 5.6 times that of 10 ms RTT flows. When the RTT ratio is more than 3 times, the throughput of long RTT flows occupy the leading position, and the bandwidth takes up about 85%. When 10 ms RTT flows coexist with 100 ms RTT flows, the fairness index of BBR is only about 0.595.

Figure 7c illustrates the throughput change and fairness index of BBQ in 0.5 BDP buffer. Compared with Figure 7a, the fairness of RTT is improved. When the RTT ratio is less than 3 times, the flows can share bandwidth well, and the fair index is about 0.998. With the increase of RTT differences, fairness decreases and long RTT flows gradually dominates. When the RTT difference is more than 6 times, the long flows occupy 60% bandwidth, and the fairness index is about 0.94. Figure 7d illustrates the throughput change and fairness index of BBQ in 5 BDP buffer. Compared with the case of 0.5 BDP, the fairness has decreased. But compared with BBR in Figure 6b, the fairness has been greatly improved. When 10 ms RTT flows coexist with 100 ms RTT flows, the fairness index of BBQ is about 0.917.

As shown in Figure 7e,f, compared with BBR and BBQ, the RTT fairness of BBR-ACW is improved. When the RTT ratio is less than 5 times, the bandwidth occupation ratio of 10 ms RTT flows can reach 45%, and the fairness index can be above 0.986 in different buffers. Even when 10 ms RTT flows competes with 100 ms RTT flows, the fairness index can be maintained at about 0.951 in 0.5 BDP buffer and 0.949 in 5 BDP buffer.

Compared with BBR and BBQ, the RTT fairness of BBR-GC is improved in different case, as shown in Figure 7g,h. In Figure 7g, compared with 10 ms RTT flows, the bandwidth of long RTT flows is more advantageous, but the bandwidth difference is smaller. Even if 10 ms RTT flows compete with 100 ms RTT flows, the bandwidth of 10 ms RTT flows is about 38.1 Mbps. Compared with BBR-ACW, the fairness of the two algorithms is basically the same, the fairness index of BBR-GC is slightly higher, which can maintain above 0.957. In Figure 7h, the buffer size becomes 5 BDP, and the fairness of BBR-GC decreases, compared with Figure 7g. When the RTT ratio is less than 5 times, the 10 ms RTT flows of BBR-GC can share the bandwidth fairly with different RTTs flows, and the bandwidth occupation ratio of 10ms RTT can reach 45%. The fairness of BBR-ACW is slightly higher than that of BBR-GC. But when RTT difference is greater than 5 times, the fairness of BBR-GC is higher than that of BBR–ACW. The fairness index of BBR-GC can keep above 0.962 when competing with different RTTs flows.

Overall, BBR-GC algorithm has a better fairness than BBR algorithm and BBQ algorithm, and compared with BBR-ACW, the fairness of BBR-GC is slightly better. The fairness index of BBR-GC is the highest among the four algorithms, and the minimum can be kept about 0.96.

### 4.2. Channel Uutilization

This part conducts some tests to measure the throughput of the flow to calculate the channel utilization of the bottleneck link. A single flow scenario is created on the NS3 simulation platform, which simulates the ideal situation of the network. The performance of the congestion algorithm is evaluated on an ideal non-congested network to show the maximum bandwidth utilization that can be achieved under optimal conditions. In this scenario, only one sender and one receiver are tested for channel utilization with random packet loss links, and buffer size is configured from 0.1 BDP to 100 BDP. At the same time, the random packet loss rate is 0% and 1%, respectively, to test the anti packet loss ability of the four algorithms. Anti packet loss ability can avoid network congestion and reduce packet loss rate. The channel utilization of all flows is calculated according to Equation (15):

(15) $$\eta =\frac{\sum_{i}bytes_{i}}{cap*duration}$$

where $bytes_{i}$ is the length of all received packets for $flow_{i}$. Cap is the bandwidth of bottleneck link, and duration is the continuous simulation running time.

**Figure 8:**
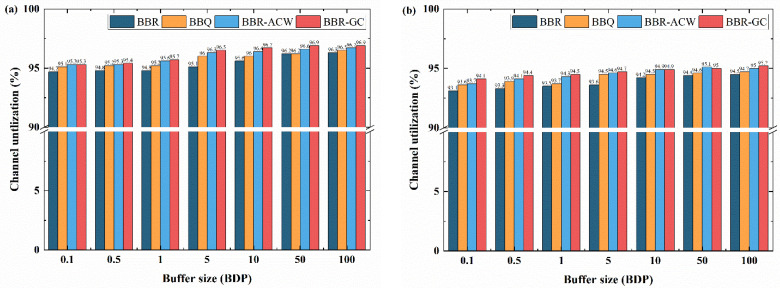
Link utilization of different congestion control algorithms: (**a**) the random packet loss rate is 0%; (**b**) the random packet loss rate is 1%.

We compared the channel utilization of BBR, BBQ, BBR-ACW and BBR-GC in different buffer sizes, and the experimental results are shown in Figure 8. Figure 8a illustrates, when the random packet loss rate is 0%, the four algorithms can achieve more than 94.7% bandwidth utilization, and BBR algorithm has the lowest channel utilization. All algorithms of BBQ, BBR-ACW and BBR-GC improve the channel utilization of BBR, especially in shallow buffers. There is no significant difference in channel utilization between BBR-ACW and BBR-GC, while BBR-GC is slightly better.

In the case where the loss rate is 1%, as shown in Figure 8b, the channel utilization of BBR-ACW and BBR-GC is higher than that of BBR and BBQ. Compared with Figure 8a, the channel utilization of BBR, BBQ, BBR-ACW, and BBR-GC are decreased, while they can still maintain more than 93.1%. Compared with BBR-ACW, the channel utilization of BBR-GC is slightly higher when buffer size is less than 5 BDP, and the difference is about 0.5%. The results show that the BBR-GC algorithm does not reduce the channel utilization, but improves the channel utilization in different buffers. Although compared with BBR-ACW algorithm, the channel utilization of BBR-GC algorithm is not significantly improved, but it is still of great significance for high-speed networks and lossy environment. In high-speed applications, even small growth can significantly improve the speed of practical application [45].

### 4.3. Retransmission

**Figure 9:**
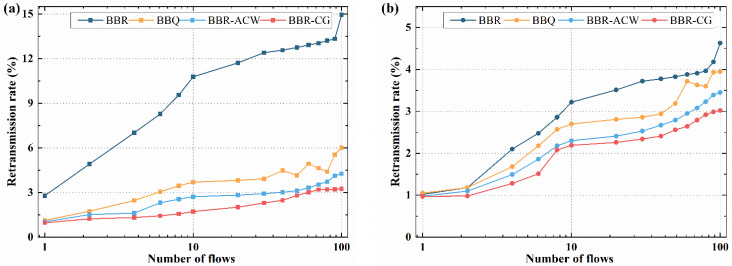
Retransmission rate for different number of RTTs flows: (**a**) 0.1 BDP buffer size; (**b**) 1 BDP buffer size.

BBR-GC algorithm introduces packet loss feedback and reduces pacing gain when the packet loss occurs. The lower the retransmission rate, the lower the datagram loss rate and the better the congestion control effect is. Therefore, we conduct experiments to verify the impact of different buffer sizes and the number of contention flows on the retransmission rate. The sender transmits the single or multiple flows using different algorithms to the receiver, and the buffer size is set to 0.1 BDP or 1 BDP. The retransmission rates are shown in Figure 9, and the starting point is the retransmission rate of a single 10 ms RTT flow.

When the buffer size is 0.1 BDP, as shown in Figure 9a, the retransmission rate of BBR is significantly higher than that of the other three algorithms. At the starting point, the retransmission rate of BBR is about 2.8%, that of BBQ is about 1.5%, and that of BBR-ACW and BBR-GC is only about 1.2%. With the increase of the number of flows, there are a lot of retransmissions in BBR. When there are 100 flows, BBR has a retransmission rate about 14.9%, while BBR-ACW and BBR-GC maintain a retransmission rate of around 4.2% and 3.2%. In summary, the retransmission rate of BBR-GC is much lower than that of BBR, and it is smaller than that of BBQ and BBR-ACW.

In Figure 9b, when the buffer size is 1 BDP, the retransmission rates of four algorithms are all lower than 0.1 BDP. When there is only one flow, the retransmission rates of four algorithms are similar, approximately 1%. When the number of flows increases to 10, BBR’s retransmission rate increases to 3.2%. Compared with BBR algorithm, BBQ reduces retransmission rate to 2.7%, BBR-ACW reduces retransmission rate to 2.5% and BBR-GC reduces retransmission rate to 2.1%. When the number of flows is 100, the retransmission rate of BBR is 4.6%, and the retransmission rate of BBQ and BBR-ACW algorithm is about 3.9% and 3.5%. BBR-GC has the lowest retransmission rate, only about 3%.

Overall, BBR-GC has the lowest retransmission rate, which has a good advantage in reducing retransmission rate. Compared with BBR algorithm, BBR-GC significantly reduces the number of retransmissions by 26% in different buffer sizes. Even compared with BBR-ACW, the retransmission rate of BBR-GC is reduced by 10%. BBR-GC adjusts the pacing rate of different RTT flows according to the link congestion state, reduce the number of retransmission and improves the efficiency of network communication.

### 4.4. Latency

**Figure 10:**
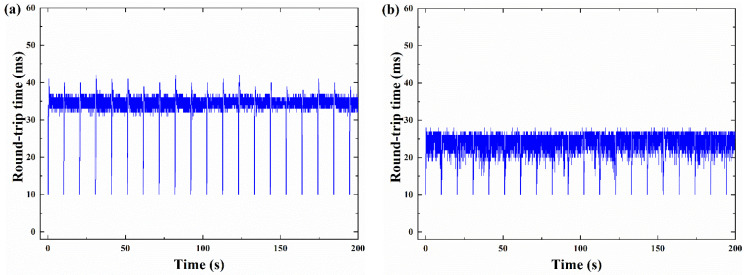

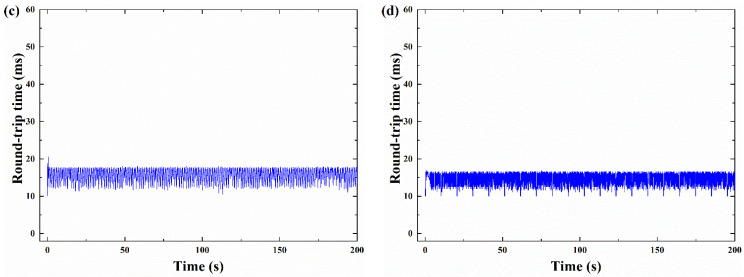
Latency of different congestion control algorithms: (**a**) BBR; (**b**) BBQ; (**c**) BBR-ACW; (**d**) BBR-GC.

In order to evaluate the effectiveness of CCAs, a latency experiment is performed. Latency is one of the main factors that cause the performance degradation or instability of network system. The smaller the delay, the faster the packet processing speed and the higher the effectiveness of congestion control. We analyze the delay statistics of the four algorithms, and design the experiment that 10 ms RTT flows competes with 50 ms RTT flows for available bandwidth at a bottleneck buffer size of 1 BDP. Figure 10 shows the latency statistics for 10 ms RTT flows with different algorithms. Figure 10a shows that the delay of BBR increases to about 35 ms. As shown in Figure 10b, the delay of BBQ is 26% lower than that of BBR, which is approximately 26 ms. Figure 10c shows, compared with the delay of the BBR and BBQ, the delay of BBR-ACW drops to about 16 ms, 54% lower than that of BBR algorithm. Figure 10d shows that the BBR-GC flow delay can be controlled around 15 ms. The average delay of BBR-GC is 57% lower than that of BBR, and lower than that of BBQ and BBR-ACW. Overall, the delay comparison results of the four algorithms show that BBR-GC can avoid the high delay caused by deep queue creation. The effectiveness of BBR-GC algorithm in congestion control is further verified.

## 5. Conclusions

This paper further analyzes the reasons for the intra-protocol RTT fairness of BBR. There is a serious deviation in bandwidth allocation, especially in deep buffer. According to the mechanism of unfairness, we propose an improved algorithm BBR-GC. BBR-GC adjusts the pacing gain (instead of fixed 1.25 or 0.75) by gamma correction function, and then adjusts the pacing rate of each flow. The coefficients of probe up and probe down of each flow are interleaved with each other, so that different RTT flows can detect bandwidth fairly.

Simulation results on NS3 show that the BBR-GC algorithm can alleviate the RTT fairness problem of BBR. Compared with BBR algorithm, BBR-GC algorithm can make multiple flows with different RTTs compete fairly. The fairness index of BBR-GC is best in different buffer sizes, which is 50% higher than that of BBR algorithm. Moreover, in the channel utilization experiments, compared with BBR algorithm, BBR-GC algorithm improves the channel utilization of BBR algorithm. Especially in shallow buffers and lossy environments, and due to active bandwidth detection, channel utilization can be greatly improved. Besides, the retransmission rate of BBR-GC is far lower than that of BBR in different buffer size, which is the lowest among the comparison algorithms in the experiments. Compared with BBQ and BBR-ACW, it has a better performance. In the latency experiment, the delay of BBR-GC algorithm is 57% lower than that of BBR, which is the lowest of the four algorithms. According to the simulation results of channel utilization, retransmission and latency, it can be seen that BBR-GC algorithm not only alleviate the RTT fairness problem of BBR flow in different periods, but also improves the effectiveness of congestion control.

In our future work, we will further optimize BBR-GC and study the optimal parameters of gamma correction. Besides, we will continue to study BBR and BBR v2, and apply the improved method to the latest BBR v2 to solve the fairness problem.

## Data Availability

Not applicable.
